# Survey of *Staphylococcus aureus* carriage by free‐living red deer (*Cervus elaphus*): Evidence of human and domestic animal lineages

**DOI:** 10.1111/tbed.14500

**Published:** 2022-03-22

**Authors:** Camilla Luzzago, Stefania Lauzi, Ralf Ehricht, Stefan Monecke, Luca Corlatti, Luca Pedrotti, Renata Piccinini

**Affiliations:** ^1^ Department of Veterinary Medicine Università degli Studi di Milano Milan Italy; ^2^ Coordinated Research Center ‘‘EpiSoMI’’ Università degli Studi di Milano Milan Italy; ^3^ Leibniz Institute of Photonic Technology (IPHT) Jena Germany; ^4^ InfectoGnostics Research Campus Jena Germany; ^5^ Institute of Physical Chemistry Friedrich‐Schiller University Jena Germany; ^6^ Institute for Medical Microbiology and Virology Dresden University Hospital Dresden Germany; ^7^ Parco Nazionale dello Stelvio Bormio Italy; ^8^ Chair of Wildlife Ecology and Management University of Freiburg Freiburg Germany

**Keywords:** cervidae, DNA microarray analysis, multi‐host pathogen, red deer, *Staphylococcus aureus*, wildlife

## Abstract

*Staphylococcus aureus* is a pathogen that can affect multiple host species. Evidence of transmission between humans and animals and among different animal species has been reported in recent years. In this study, we investigated 284 free‐living red deer (*Cervus elaphus*) in the Central Italian Alps to assess the prevalence and molecular characteristics of *S. aureus* in nasal and intestinal samples in relation to host features and environmental factors. A prevalence of 90%, 26.2% and 10.7% of *S. aureus* was detected in nasal rectal swabs and faeces, respectively. Calves had a higher probability of being *S. aureus* intestinal carriers than adults, especially in females when considering faecal samples. Clonal complex (CC) 425 was the most prevalent lineage (61.5%). This is a lineage known to be widespread in both domestic and free‐living animals. It was followed by CC2671 (15.4%) and CC350 (6.4%). A high rate of the phage‐borne virulence factor *lukM/lukF‐P83* was detected in CC425 and CC350. Further lineages, which are known to occur in both humans and animals, were detected sporadically in red deer faeces only, that is, CC7, CC9, CC121 and CC707, harbouring the genes of the penicillinase operon and a gene for macrolide resistance (CC9 and CC121). Methicillin resistance genes *mec*A and *mec*C were not found. Our results suggest that free‐living red deer may be reservoir for *S. aureus* in Alpine habitats.

## 1 INTRODUCTION

*Staphylococcus aureus* is a generalist bacterial species that inhabits mucosae and skin in humans and animals (Haag et al., 2019; Tong et al., 2015), and whose primary ecological niche is found in the nares.

*Staphylococcus aureus* is frequently detected in healthy carriers but, as an opportunistic pathogen, it can cause a number of infections ranging from superficial skin diseases to deep or generalized infections and septicaemia (Lowy, 1998; Peton & Le Loir, 2014). After the introduction of the first antibiotics, *S. aureus* revealed an ability to acquire various mechanisms of resistance and, for instance, methicillin‐resistant *S. aureus* (MRSA) strains were isolated few years after introduction of this compound (Jevons, 1961). In livestock, *S. aureus* is a major causal agent of mastitis, a disease that greatly affects the economics of dairy cattle and small ruminant industries (Bergonier et al., 2003; Ruegg, 2017). *Staphylococcus aureus* transmission events between humans and livestock have been described and the massive use of antibiotics in both, human and veterinary medicine, might provide a selective pressure favouring antibiotic resistance (Haag et al., 2019; Richardson et al., 2018). The role of other host species is still less clearly defined. A wide collection of strains of *S. aureus* from European wildlife, including cervids, has recently been investigated, highlighting a great genetic diversity and rather low antimicrobial resistance rates (Monecke et al., 2016). The occurrence of MRSA in wildlife without exposure to antibiotics has been recently reviewed (Heaton et al., 2020: Silva et al., 2020), showing that most MRSA isolated from wild animals are *mec*C positive. The dissemination of this mechanism of resistance (García‐Álvarez et al. et al., 2011; Shore et al., 2011) in free‐living animals and river waters (Porrero et al., 2014a) suggests that the natural environment may be a reservoir of *mec*C gene, and indeed, a co‐colonisation of hedgehogs with *S. aureus* and dermatophytes that produce penicillin provides a selective pressure favouring *mecC* in the wild (Dube et al., 2021; Larsen et al., 2022; Smith & Marples, 1965).

Red deer (*Cervus elaphus*) populations are currently increasing in density and distribution across Europe (Lovari et al., 2018), also owing to the ecological plasticity that allows this species to inhabit both natural environments such as forests or pastures, as well as agricultural landscapes. The interest to acquire baseline information on untreated free‐living animals and monitor the possible effect of the influx of human‐ and livestock‐associated *S. aureus*/MRSA on wildlife has been recognized (Heaton et al., 2020; Monecke et al., 2016).

The aim of this study was focused on an Alpine population of free‐living red deer to systematically survey the *S. aureus* population structure to evaluate the role of red deer in *S. aureus* epidemiology, in relation to host features and environmental factors.

## 2 MATERIALS AND METHODS

### 2.1 Study population and area

The red deer study population inhabits the Central Italian Alps in the Stelvio National Park. A large increase in red deer density occurred over the past decades (up to some 31 individuals/km^2^ in wintering areas), leading the Park to start a culling program in 2011, with the aim to reduce the red deer density as well as its impact on agricultural activities, forest regeneration and biodiversity. Between 2014 and 2017, annual surveys resulted in an estimation of the deer population within the Park of approximately 1800 individual animals, with a mean density of 9.5 individuals/km^2^ in autumn and 27.4 ind./km^2^ in winter (Corlatti et al., 2016; Pedrotti et al., 2017).

Other wild ungulates in the area include roe deer (*Capreolus capreolus*), chamois (*Rupicapra rupicapra*) and ibex (*Capra ibex*). Furthermore, herds of cattle and small domestic ruminants share alpine pastures with red deer in summer.

The study area corresponds to the red deer wintering site within the Province of Sondrio and ranges between 1200 and 2400 m a.s.l. The study area was functionally divided into three macroareas with different levels of anthropization (low, medium, high) (Formenti et al., 2015; Lauzi et al., 2021). Information on land use were retrieved from CORINE land cover maps (https://land.copernicus.eu/pan‐european/corine‐land‐cover) and processed with QGIS (https://qgis.org/it/site/) to define the proportion between the surface occupied by human settlements/farming activities and the whole surface of the culling units. Human settlements and agricultural landscapes around small villages constitute 7% of the whole surface in the low anthropized macroarea (772 ha), whereas they represent 32% of the total surface in the high anthropized macroarea (707 ha). A total of 1200 ha are considered medium anthropized macroarea. Observations on radio‐tracked animals suggest limited deer movements among macroareas in autumn and winter.

### 2.2 Sampling and data collection

Red deer were annually culled by hunters in winter, under the supervision of the Park Authority. The culling plan was legally authorized by ISPRA, the Italian Ministry of Environment (Prot. 48585/T‐A25‐Ispra). The present study was carried out in two consecutive seasons (2016–2017 and 2017–2018). Within a few hours after culling, animals were brought to the control centre of the Park, where individual information including age, sex, body mass and culling area were registered. Animals were classified as calves (<1‐year old), yearlings (1‐year old) and adults (≥ 2years old). Nasal and rectal swabs were collected using Amies Agar Gel Transport Swab (Oxoid, Fisher Scientific Italia, Rodano, Italy). Swabs were taken with care to avoid touching the skin and were put directly into the transport tube to avoid cross‐contamination. Faeces were taken, using disposable gloves, directly from the rectum and put into a sterile container. In the first season, nasal swabs, rectal swabs and faeces were sampled, whereas in the second season, only nasal swabs and faeces were collected. Specimens were stored at the check point at −20°C. Subsequently, frozen samples were transferred to the laboratory monthly for further processing. A preliminary analysis, processing two replicates of samples after 1–2 days and after 28 days of storage at –20°C showed the same *S. aureus* detection rate (data not shown).

### 2.3 *Staphylococcus aureus* isolation

Nasal swabs were incubated in 5 ml of Mueller‐Hinton broth with 6.5% NaCl at 37°C for 24 h. Pre‐enrichment cultures were subsequently plated on Baird‐Parker with rabbit plasma fibrinogen (Oxoid Baird‐Parker RPF Agar Base, Fisher Scientific Italia, Rodano, Italy) and incubated for 48 h at 37°C. Faeces were suspended in sterile H_2_O (≅ 1 g/sample in 4 ml), diluted (1:10) in Mueller‐Hinton broth with 6.5% NaCl and then processed as previously described for swabs. One to four grey/black colonies with an opaque halo per sample were isolated on Blood Agar plates (Microbiol Diagnostici, Cagliari, Italy). Bacterial colonies were identified as *S. aureus* by colony morphology, haemolysis pattern and coagulase reaction, and the identification was confirmed by amplification of the *nuc* gene (Baron et al., 2004).

### 2.4 DNA microarray analysis

For each animal, one nasal *S. aureus* colony and two rectal or faecal colonies were analyzed in the first sampling season and two faecal colonies in the second sampling season. Specifically, *S. aureus* isolates were selected from animals that were positive at both, nasal and intestinal sites by rectal swab or faeces and from faecal carriers, irrespective of nasal status. *Staphylococcus aureus* isolates were characterized using the StaphyType DNA microarray based assay (Alere Technologies, Jena, Germany). This assay detects *S. aureus* target sequences, including species and antimicrobial resistance markers, allowing isolates to be assigned to MLST sequence types (STs) and/or clonal complexes (CCs), and staphylococcal cassette chromosome *mec* (SCC*mec*). Details of these procedures as well as a list of target sequences have been previously described in Monecke et al. (2008).

### 2.5 Statistical analysis

A Paerson's chi‐square test was used to assess the relationship between presence of *S. aureus* and independent variables such as type of sample, season of collection, sex, age and anthropization level. A *p*‐value < .05 was considered as statistically significant, whereas tendency was considered in the presence of *p*‐value >.05 but <.1. Statistical analysis was performed using Epitools‐Epidemiological calculators available at: http://epitools.ausvet.com.au (Sergeant, 2018).

Furthermore, generalized linear models with binomial error distribution and logit‐link were fitted to investigate the effect of several predictors on different response variables, modelled as Bernoulli variables (presence: ‘1’; absence: ‘0’). Specifically, we investigated the effect of age class (calves, yearling and adults), sex, body mass, anthropic level (low, medium, high) on (i) the probability of presence of *S. aureus* in nasal swabs; (ii) the probability of presence of *S. aureus* in rectal swabs; (iii) the probability of presence of *S. aureus* in the faeces; (iv) the probability of presence of ‘human‐livestock type’ *S. aureus* in rectal swabs and faeces. In ‘human‐livestock type’ *S. aureus*, we grouped CCs previously reported in both humans and livestock, that is, CC7, C9, CC121, CC350 and CC707. Prior to data analysis, due to the paucity of data, we grouped the predictor levels yearlings and adults as well as low and medium anthropic level into one level, defined as ‘adults’ and ‘medium’, respectively. Preliminary data exploration revealed multicollinearity between age class and body mass (variance inflation factor values > 3; Zuur et al., 2010) and we thus removed body mass from subsequent modelling. For each response variable, we first fitted a beyond optimal model where the linear predictor included sex in interaction with age class and anthropic level, except for ‘human‐livestock type’ *S. aureus*, where all terms were additive due to paucity of data. Next, stepwise selection was performed to find a more parsimonious model.

We inspected the adequacy of each final model through quantile residual diagnostics (Dunn & Smyth, 2018), assessed the Tjur's coefficient of discrimination (Tjur, 2009) and calculated the predictive accuracy by means of the area under the receiver operating characteristic (AUC‐ROC) curve (Fawcett, 2006). Given the small sample size, robust estimates of confidence intervals were obtained by bootstrap using 1000 iterations and the adjusted percentile method.

All analyses were conducted with R v. 4.0.4 (R Core Team, 2020) in RStudio v. 1.3.1056 (R Studio Team, 2020) using the following packages: ‘car’ (Fox & Weisberg, 2019) for VIF inspection, ‘MASS’ (Venables & Ripley, 2002) for stepwise selection, ‘DHARMa’ (Hartig, 2020) for residual diagnostics, ‘parameters’ (Lüdecke et al., 2020a) for bootstrap estimation, ‘performance’ (Lüdecke et al., 2020b) for pseudo‐*R* ^2^, ‘pROC’ (Robin et al., 2011) for ROC values, ‘visreg’ (Breheny & Burchett, 2017) and ‘ggplot2’ (Wickham, 2016) for visualization of marginal effects.

## 3 RESULTS

### 3.1 *Staphylococcus aureus* prevalence

A total of 284 red deer were sampled to investigate characteristics of *S. aureus* nasal and/or intestinal carriage. The descriptive results of *S. aureus* prevalence are summarized in Table 1. A *S. aureus* nasal prevalence of 90 % (95% CI: 85.8‐93.0) was detected, with a slight variation between 2016–17 (91.3%) and 2017–18 (89%) culling seasons. A *S. aureus* intestinal prevalence of 26.2 % (95% CI: 18–37.6) and 10.7% (95% CI: 7.2–15.5) were reported from rectal swab and faecal samples, respectively. The highest prevalence rates were observed in calves both at nasal and intestinal sites, showing 93.3%, 46.7% and 19.4% at nares, rectal mucosa and faeces, respectively. Fifty‐one red deer were simultaneously sampled by nasal swab, rectal swab and faeces. In these animals, a higher percentage of *S. aureus* nasal carriage (88.2%) was observed compared to rectal (23.5%) or faecal carriage (5.9%). Moreover, *S. aureus* was detected only from nares in 60.8%, from nasal and rectal mucosae simultaneously in 21.6% and from nares and faeces in 3.9%.

**TABLE 1:** *S*taphylococcus *aureus* prevalence according to culling season, age, sex, type of sample and anthropization level of the Stelvio National Park

			Positive/No. of type of sample (%)		
Variable	Category	Positive/No. of animal (%)	Nasal swab	Rectal swab	Faeces
Culling season	2016–2017^†^	106/118 (89.8)	105/115 (91.3)	16/61 (26.2)	6/70 (8.6)
	2017–2018	125/166 (75.3)	138/155 (89.0)	–	17/145 (11.7)
Age	<1‐year old	75/88 (85.2)	70/75 (93.3)	7/15 (46.7)	13/67 (19.4)^a,b^
	1‐year old	46/51 (90.2)	45/51 (88.2)	0/8 (0)	1/35 (2.9)^a^
	≥2 years old	130/145 (89.7)	128/144 (88.9)	9/38 (23.7)	9/113 (8.0)^b^
Sex	Female	150/171 (87.7)	147/162 (90.7)	11/45 (24.4)	11/135 (8.1)
	Male	101/113 (89.4)	96/108 (88.9)	5/16 (31.3)	12/80 (15.0)
Anthropization level	Low	68/74 (91.9)	68/74 (91.9)	1/12 (8.3)	4/49 (8.2)
	Medium	69/81 (85.2)	66/78 (84.6)	10/25 (40.0)	6/60 (10.0)
	High	114/129 (88.4)	109/118 (92.4)	5/24 (20.8)	13/106 (12.3)
Total		231/284 (81.3)	243/270 (90)^a,b^	16/61 (26.2)^a,c^	23/215 (10.7)^b,c^

^†^ Data partially from Luzzago et al. (2019).

^a,b,c^Significant difference between categories of the same variable (*p* < .05).

### 3.2 Microarray based assays

A total of 79 *S. aureus* isolates obtained from 30 red deer were analyzed (Data set 1 in the Supporting Information). Eight CCs, namely CC7, CC9, CC121, CC350, CC425, CC707, CC2328 and CC2671, were identified (Table 2). CC425 was the most prevalent lineage (60.8%), followed by CC2671 (16.5%) and CC350 (6.3%). Only these three predominant lineages were detected at both nasal and rectal mucosa, whereas the other lineages (CC7, CC9, CC121, CC707) were identified sporadically and in faeces only. Faecal samples showed the highest *S. aureus* genetic diversity with seven CCs detected. The presence of resistance and virulence genes was summarized in Table 2. Notably, 68.7 % of CC425 strains and 60% of CC350 carried the leukocidin genes *lukM/lukF‐P83*, which are not typical for these lineages but frequently associated with other CCs isolated from bovine or small ruminant udder infections, that is, CC151, CC479 and CC133 (Hoekstra et al., 2020; Schlotter et al., 2012). The genes encoding for Panton‐Valentine leukocidin (PVL) were not found in this study. *Sak* and *scn* genes were detected in CC7, CC9, CC121 and CC707 isolates, while the *chp* gene was found in CC9 and 707. Regarding enterotoxins, CC121 harboured the *seb* gene.

**TABLE 2:** *S*taphylococcus *aureus* isolates characterized using DNA microarray analysis

	Frequency by sample type				Resistance genes				Virulence genes					
Clonal complex	Total No. (%)	Nasal swab No. (%)	Rectal swab No. (%)	Feces No. (%)	*blaZ*	*blaI*	*blaR*	*ermA*	*lukM* No. (%)	*lukF‐P*83 No. (%)	*seb*	*sak*	*chp*	*scn*
CC7	5 (6.3)	0	0	5 (16.6)	+	+	+	–	–	–	–	+	–	+
CC9	2 (2.5)	0	0	2 (6.7)	+	+	+	+	–	–	–	+	+	+
CC121	3 (3.8)	0	0	3 (10.0)	+	+	+	+	–	–	+	+	–	+
CC350	5 (6.3)	1 (5.0)	2 (6.9)	2 (6.7)	–	–	–	–	3 (60)	3 (60)	–	–	–	–
CC425	48 (60.8)	15 (75.0)	23 (79.3)	10 (33.3)	–	–	–	–	33 (68.7)	33 (68.7)	–	–	–	–
CC707	2 (2.5)	0	0	2 (6.7)	+	+	+	–	–	–	–	+	+	+
CC2671	13 (16.5)	3 (15.0)	4 (13.8)	6 (20.0)	–	–	‐	–	–	–	–	–	–	–
CC2328	1 (1.3)	1 (5.0)	0	0	–	–	–	–	–	–	–	–	–	–

*Note*:+: All isolates of the respective CC were positive; –: all isolates of the respective CC were negative.

All isolates were tested for methicillin resistance genes *mec*A and *mec*C but all were negative. CC7 and CC707 harboured the genes of the penicillinase operon (*blaZ/I/R*); CC9 and CC121 were multiresistant, carrying also a gene for macrolide resistance (*ermA*).

Strain typing showed in 15 out of 20 animals investigated a presence of the same CC at both sampling sites (Table 3). In detail, 12 animals harboured CC425, two CC2671 and one CC350. Concerning the two *S. aureus* isolates analyzed per sampled site, the same CC was always observed for both isolates from rectal mucosa, whereas in colonies from faeces a different CC was identified in four out of 14 cases.

**TABLE 3:** *Staphylococcus aureus* clonal complex by animal and types of sample

					Rectal swab		Feces	
Animal ID	Sampling data	Age	Sex	Nasal swab	First colony	Second colony	First colony	Second colony
806	28/01/2017	≥2 years old	f	CC425	–	–	CC707	CC707
802	28/01/2017	≥2 years old	f	CC425	CC425	CC425	–	–
794	29/01/2017	≥2 years old	f	CC2328	CC425	CC425	–	–
628	05/02/2017	≥2 years old	f	CC425	–	–	CC7	CC425
555	07/02/2017	≥2 years old	f	CC425	CC425	CC425	n/a	n/a
556	07/02/2017	1‐year old	m	CC425	CC425	CC425	–	–
482	11/02/2017	≥2 years old	f	CC425	CC425	CC425	–	–
837	12/02/2017	≥2 years old	m	CC425	CC425	CC425	n/a	n/a
485	14/02/2017	≥2 years old	m	CC425	CC425	–	–	–
824	14/02/2017	≥2 years old	f	CC425	CC2671	CC2671	–	–
829	14/02/2017	1‐year old	m	CC425	CC425	CC425	–	–
701	16/02/2017	1‐year old	m	CC425	n/a	n/a	CC425	CC425
831	16/02/2017	1‐year old	f	CC425	CC425	CC425	–	–
314	18/02/2017	1‐year old	m	CC425	n/a	n/a	CC9	CC9
816	18/02/2017	1‐year old	m	n/a	–	–	CC350	CC350
767	21/02/2017	1‐year old	f	CC2671	+	+	CC2671	–
757	21/02/2017	1‐year old	f	CC2671	CC2671	CC2671	–	–
721	23/02/2017	≥2 years old	f	CC2671	CC425	CC425	n/a	n/a
711	23/02/2017	1‐year old	m	CC425	CC425	CC425	n/a	n/a
581	26/02/2017	1‐year old	f	CC425	CC425	CC425	–	–
704	26/02/2017	≥2 years old	f	CC350	CC350	CC350	–	–
593	09/01/2018	≥2 years old	m	–	n/a	n/a	CC2671	CC2671
878	09/01/2018	≥2 years old	f	+	n/a	n/a	CC425	CC425
882	11/01/2018	1‐year old	f	+	n/a	n/a	CC425	CC121
869	13/01/2018	1‐year old	m	+	n/a	n/a	CC121	CC121
892	14/01/2018	1‐year old	f	+	n/a	n/a	CC2671	CC2671
918	06/02/2018	1‐year old	m	n/a	n/a	n/a	CC7	CC7/CC2671
933	06/02/2018	1‐year old	f	n/a	n/a	n/a	CC7	CC425
975	08/02/2018	1‐year old	f	n/a	n/a	n/a	CC425	CC425
977	08/02/2018	1‐year old	m	n/a	n/a	n/a	CC425	CC7

*Note*: n/a: sample not available; +: *S. aureus* positive, microarray not performed.

### 3.3 Statistical analysis

**FIGURE 1:**
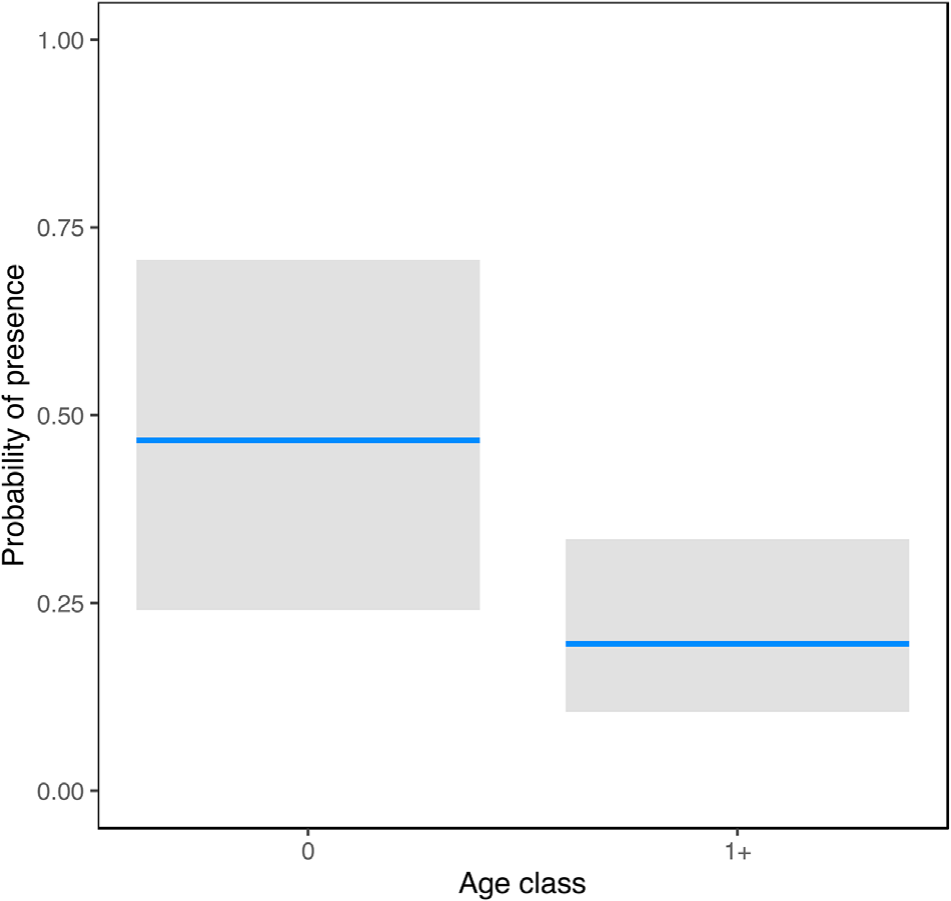
Marginal effects of age class (calves [0] and adults [1+]) to explain variation in the probability of presence of *S. aureus* in rectal swabs

**FIGURE 2:**
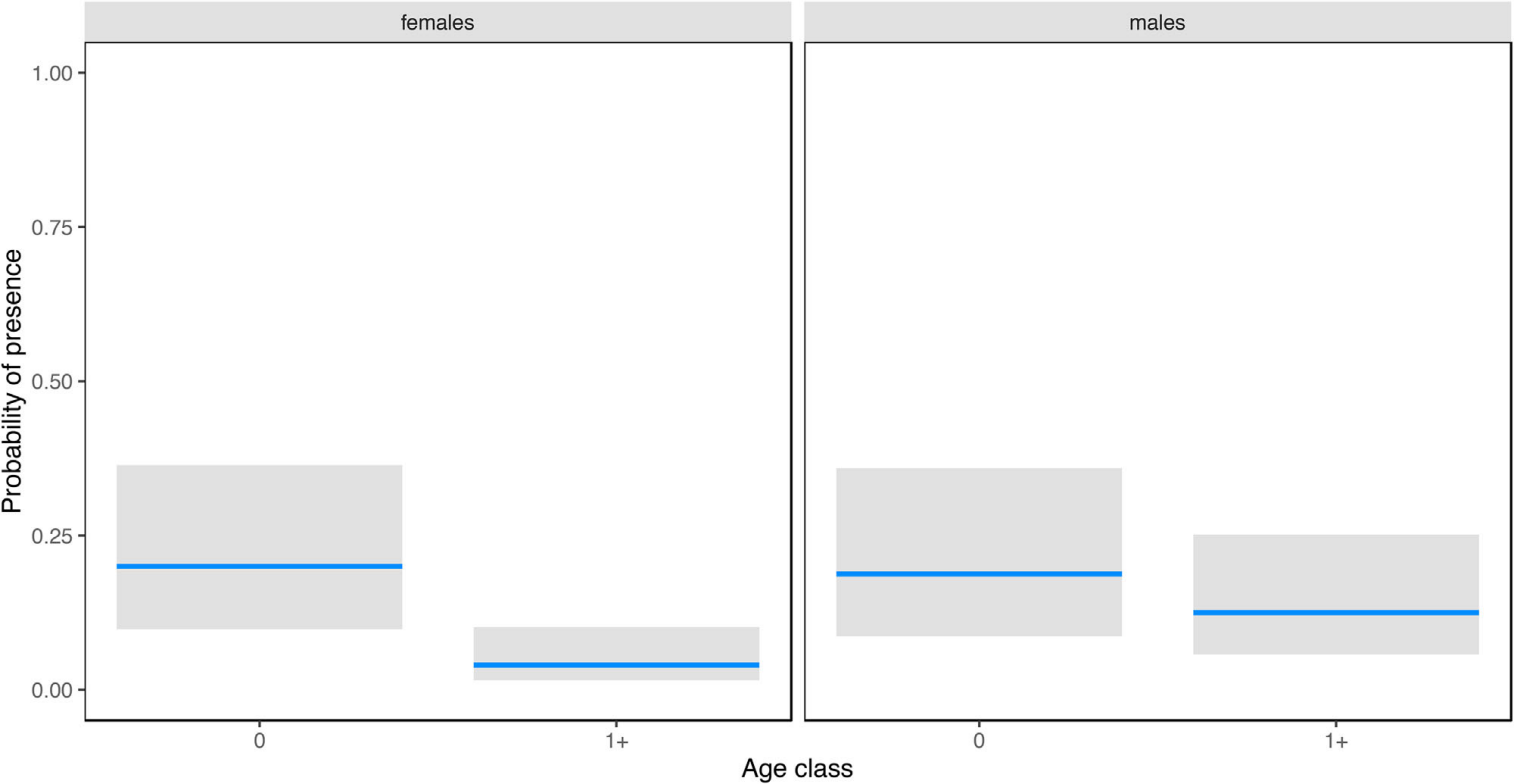
Marginal interactive effects of age class (calves [0] and adults [1+]) by sex (males and females) to explain variation in the probability of presence of *S. aureus* in faeces

*Staphylococcus aureus* positivity rate was statistically different among types of sample (*p* < .0001), with a higher number of positive nasal swabs compared to other types (*p* < .0001 vs. rectal swabs, and *p* < .0001 vs. faeces). A higher number of positive samples were also significantly observed in rectal swabs compared to faeces (*p* = .0042). Nasal swabs were confirmed as the sample with the highest significant positivity rate compared to rectal swabs (*p* < .0001) and faeces (*p* < .0001) in the 51 red deer with the three types of samples collected simultaneously. In this subgroup of 51 animals, faeces were the sample with the significantly lowest positivity also compared to rectal swabs (*p* = .0253). Positivity of *S. aureus* in rectal and faecal samples was statistically different among age groups (*p* = .0448 and *p* = .0146, respectively) but no differences were observed for nasal swabs positivity in the different age groups. Calves showed a significantly higher *S. aureus* faecal positivity rate compared to yearlings (*p* = .0452) and adults (*p* = .0424). The positivity rate tended to be higher in rectal swabs from calves compared to yearlings (*p* = .0656). The statistical analyses showed no significant association of *S. aureus* positivity with season of collection, sex or anthropization level. After the stepwise selection, the probability of presence of *S. aureus* in nasal swabs was best explained by a ‘null’ model, that is, an intercept‐only model with mean probability of 90%, suggesting that none of the predictors explained the variation in the response variable (Table 4). The variation in the probability of the presence of *S. aureus* in rectal swabs was best explained by a model that included only age class as a predictor (pseudo‐*R* ^2^ = 7%, AUC‐ROC = 0.63). The regression coefficient returned an odds‐ratio of *e* ^(−1.291)^ = 0.27, suggesting a change, albeit non‐significant, of −73% in the odds of *S. aureus* presence from calves to adults, that is a mean probability of presence of *S. aureus* of 47% in calves and 20% in adults (Table 4, Figure 1). The probability of presence of *S. aureus* in the faeces was best explained by the interactive effect of sex and age class (pseudo‐*R* ^2^ = 5%, AUC‐ROC = 0.69). Although the interaction was non‐significant, the effect size was non‐negligible, suggesting that the mean probability of presence of *S. aureus* from calves to adults decreased more rapidly in females (from 20% to 4%) than in males (from 19% to 13%) (Table 4, Figure 2). The variation in the probability of the presence of ‘human‐livestock type’ *S. aureus* in the faeces and rectal swabs was also explained by a ‘null’ model, with a mean probability of presence of 27% (Table 4). All the selected models adequately described the data, as suggested by the unsystematic distribution of quantile residuals. For rectal swabs and faecal samples, the values of the area under the ROC curve showed limited predictive accuracy.

**TABLE 4:** Model estimates for the effects of different predictors selected to explain variation in (i) the probability of presence of *S. aureus* detected from nasal swabs; (ii) the probability of presence of *S. aureus* detected from rectal swabs; (iii) the probability of presence of *S. aureus* detected from faeces; (iv) the probability of presence of ‘human‐livestock type’ *S. aureus* detected from faeces and rectal swabs. The table reports estimates of regression coefficients, lower and upper bounds of 95% bootstrap confidence intervals (2.5% and 97.5% quantiles)

Parameters	Estimate	95% Bootstrap confidence interval	
		2.5% quantile	97.5% quantile
*S. aureus from nasal swabs*			
(Intercept)	2.197	1.800	2.595
*S. aureus from rectal swabs*			
(Intercept)	−0.134	−1.386	1.099
Age class (adults vs. calves)	−1.291	−2.670	0.049
*S. aureus from fecal samples*			
(Intercept)	−1.386	−2.639	−0.606
Sex (males vs. females)	−0.065	−1.529	1.291
Age class (adults vs. calves)	−1.822	−3.574	−0.396
Sex (males vs. females): Age class (adults vs. calves)	1.406	−0.856	3.688
*‘Human.livestock type’ S. aureus*			
(Intercept)	−1.012	−1.821	−0.202

## 4 DISCUSSION

Our study allowed us to assess the prevalence and genetic diversity of *S. aureus* in a population of free‐living red deer in the Alps, identifying the frequency of carriers, genetic lineages and resistance genes at nasal and intestinal sites also in relation to host and environmental factors. The prevalence of *S. aureus* was particularly high in nares, with a carriage rate higher than those previously reported in free‐living red deer, that is, 90% in this study versus values between 19.2% in Spain (Porrero et al., 2014b) and 49% in Germany (Meyer et al., 2014). The *S. aureus* isolation protocol applied in this study, with enrichment and selective steps not or partially reported in the above investigations, may have contributed to an increase in the detection rates. The high population density of red deer, reaching a winter density of nearly 30 ind./km^2^ in the study area (Corlatti et al., 2016), however, should be also considered, as it is likely to increase the transmission of *S. aureus* by direct contact, by ingestion of contaminated forages on pasture or by inhalation of infected droplets (Kozajda et al., 2019). It is difficult to compare results of *S. aureus* carriage with respect to density of red deer population because densities are subjected to annual fluctuations and different management strategies may occur in Europe, including reintroduction programs (Gnat et al., 2015).

In the present study, the rate of intestinal carriers is significantly lower than the one of nasal carriers both from rectal swabs (26.2%) and faeces (10.7%). A similar trend was reported in pigs (Khanna et al., 2008) and humans, where the average detection rate of intestinal carriage is 20% in healthy individuals and patients (Acton et al., 2009). In the present study, the significantly lower rate of intestinal *S. aureus* prevalence was also confirmed in the subset of red deer simultaneously sampled by nasal and rectal swabs and faeces.

The literature on *S. aureus* detection at intestinal site in red deer is scant, although that issue might be of relevance with regard to transmission. To the best of our knowledge, there are only two studies addressing this issue and they detected *S. aureus* in 0.67 % of faecal samples collected in the free‐living population from Hungary, Slovakia and Poland (Gnat et al., 2015) and in 33.3% from Spain (García et al., 2020).

Noteworthy is the higher probability of calves being *S. aureus* intestinal carriers, compared to adults, with regard to both rectal mucosa and faeces. Even though some caution is required in the interpretation of the statistical results, owing to the limited sample size, this age‐related pattern is not surprising, as colonization of the intestinal tract with *S. aureus* in newborns and young children is observed at a high frequency also in humans, suggesting the occurrence of a mechanism of mother‐to‐child transmission (Acton et al., 2009). Calf behaviour with direct contact with mother, suckling and closeness among individuals likely explains the higher prevalence observed compared to adults. Moreover, lactation in red deer is long termed and it could last until 12 months of age in barren females (Clutton‐Brock et al., 1982) and occasional allosuckling, that is, suckling from a non‐maternal hind, has been reported in the wild (Bartoš et al., 2001). Although we did not perform analysis on milk samples, long‐term suckling and skin contact may have contributed to an increase in the probability of infection in calf. The potential sex‐related variation in the probability of presence of *S. aureus* in faeces from calves to adults, greater in females than in males, however, still remains unclear, though it might be related to a higher susceptibility to *S. aureus* of adult males compared to adult females, possibly due to the great energy expenditure of the former during rutting season.

Within the subset of *S. aureus* isolates analyzed by microarray, CC425 was the most prevalent lineage in all types of samples, reaching similar rates in the nares and rectal mucosa, 75% and 79.3%, respectively, and 33.3% in faeces. This lineage was previously identified in red deer in Austria, Germany and Spain (Porrero et al., 2014b; Monecke et al., 2016) and it is known to be widespread both in domestic and wild animals (Monecke et al., 2016). Differently from previous isolates, more than half of CC425 detected in our study carried the genes for *lukM/lukF‐P83*, a leukocidin similar to the PVL (Kaneko & Kamio, 2004) and associated with bovine mastitis (Herron‐Olson et al., 2007; Schlotter et al., 2012). Cross‐infection might pose a risk to domestic animals, even though some caution is required in the interpretation of this finding. Indeed, the *lukM/lukF‐P83* positive lineages that have been reported in domestic cattle are others (CC151, CC479) than the ones in deer (Hoekstra et al., 2018).

CC425 is one of the lineages that have been observed to harbour SCC*mec* XI elements including the *mec*C gene (García‐Alvarez et al., 2011). This, however, was not the case in our isolates.

In the present study, CC2671 and CC350 were also identified in the nares, as well as in the rectum and faeces, but at much lower extent compared to CC425. CC2671 has sporadically been observed in red deer in Spain (Porrero et al., 2014b), and there are two entries of isolates from Spanish red deer in the MLST database (https://pubmlst.org/bigsdb?page = info&db = pubmlst_saureus_isolates&id = 5037 and 5038). CC350 is rarely reported in humans and cattle (Monecke et al., 2016). Strain typing suggested involvement of the same strains for both nasal and rectal mucosae. Genes *lukM/lukF‐P83* were also found in CC350 isolates, another group in which they have, to the best of our knowledge, not yet been observed or where they are rare or where they might be restricted to host‐specific lineages from animals that not yet have systematically been studied. The high rate of the phage‐borne virulence factor *lukM/lukF‐P83* in red deer isolates (36 out of 79 isolates tested) suggests that it might play a pathogenetic role in *S. aureus* from red deer. It is beyond the scope of the present study to prove that experimentally, but further studies should aim at its role in infection or disease in red deer. It should be considered that the finding of the same CC or specific virulence factors in free‐living animals as in humans and livestock is not evidence of an interspecies transmission event.

Further lineages were identified in faecal samples and included some that are known to infect humans and animals. In particular, CC7 has been reported in humans and cattle (Conceição et al., 2017; Cuny et al., 2019; Monecke et al., 2016). CC9 is recognized as a livestock‐associated lineage in domestic pigs and farm personal (Mroczkowska et al., 2017; Ye et al., 2016), CC121 has been reported both in humans, in whom it is widespread and common, and rabbits (Kurt et al., 2013; Vancraeynest et al., 2006) and CC707 sporadically in humans (Egyir et al., 2014) and animals. The above‐mentioned lineages were never reported in cervids, with the exception of CC707 that once was identified in reindeer (*Rangifer tarandus*) in Sweden (Monecke et al., 2016). CC2328 is too rare and poorly known so no assessment on geographic distribution or natural hosts can be provided. There is one genome sequence from Spain stored in the NCBI database (SAMEA2298588) but no details are provided. In addition, there is one isolate from Danish sheep described in the MLST database (https://pubmlst.org/bigsdb?page = info&db=pubmlst_saureus_isolates&id=4505) as well as three further genomes without epidemiological data.

In the present study, *S. aureus* lineages showed a higher genetic diversity in faeces compared to nasal and rectal mucosae in the red deer population of the Stelvio National Park with CC7, CC9, CC121 and CC707 detected only in the faecal specimen. In contrast, CC425, CC2671 and CC350 were frequently isolated at nasal and rectal mucosae and faeces. This could suggest that mechanisms for nasal and intestinal carriage could differ from that of faeces. Nasal and rectal mucosa may be susceptible to specific variants of *S. aureus* closely associated with red deer and more proficient colonizers than others, while a *S. aureus* passive shedding through faeces could be hypothesized due to red deer exposure to several *S. aureus* lineages during feeding. These hypotheses need to be explored by combining the preliminary screening by microarray‐based methods with genotyping methods with a high discrimination power, such as whole‐genome sequencing. Human settlements and agricultural landscapes were considered as environmental predictors of exposure to human‐livestock lineages of *S. aureus*, but no statistically significant relationship has been observed in this study.

Despite the extensive typing of *S. aureus* isolates, the other most prevalent lineages reported in wild ruminants in several European countries, namely CC97, CC130 and CC133 (Monecke et al., 2016), were not detected in our study area. This result was unexpected because CC97 and CC130 have been reported in Western Italian Alps in roe deer and chamois (Luzzago et al., 2014) and might also represent livestock‐associated lineages in Italy (Feltrin et al., 2016; Romanò et al., 2020).

Notably, no MRSA isolates were detected, supporting previous findings on free living red deer in Europe that reported absence or sporadic occurrence of MRSA (Monecke et al., 2016; Porrero et al., 2013, 2014b). Nevertheless, CC7, CC9, CC121 and CC707 lineages harboured penicillin resistance genes and CC9 and CC121 also a macrolide resistance gene (*ermA*). These findings highlight that resistance genes are restricted to *S. aureus* CCs that are known to infect and or colonize both humans and domestic animals supporting the hypothesis of red deer exposure to an environment contaminated by livestock and human activities. Similarly, certain virulence genes, that is, *sak, chp* and *scn*, that are usually associated with isolates from humans, rather than with those from ungulates, might indicate exposure to human strains of *S. aureus* (Rohmer & Wolz, 2021).

Overall, our findings confirmed that nasal samples are the best option to detect *S. aureus* carriers in red deer compared to rectal mucosa and faeces, in accordance with previous results based on nares and skin (Porrero et al., 2014b). Nevertheless, although the nasal site is usually sampled to isolate *S. aureus* both from humans and animals due to the higher rate of detection compared to other sampling sites, *S. aureus* higher clonal complex diversity in faecal specimens versus nasal and rectal mucosa has to be considered in sampling dealing with *S. aureus* genetic investigation. Moreover, from an epidemiological standpoint, faeces represent an important route of dissemination of bacteria into the environment and analysis may be extended to other pathogens. Noteworthy, the present red deer population has also been reported as the carrier of potentially zoonotic Shiga toxin‐producing *Escherichia coli* (Lauzi et al., 2021).

Our results suggest that free‐living red deer may be a reservoir for *S. aureus* in Alpine environment. The *S. aureus* population mainly comprised lineages already known to occur in domestic or wild ruminants, although there were lineages on which no sufficient data are available. In general, more typing, including whole‐genome sequencing, on cervid isolates should be performed in order to identify host‐specific lineages or host‐specific factors and to understand which lineages might be endemic or involved in localized outbreaks. Human and other animal lineages, that harboured penicillin and macrolide resistance genes, were sporadic but may represent a source for environmental contamination. Untreated wild host populations, that do not experience selective pressure for resistance, could act as a reservoir for antimicrobial susceptible pathogens and may contribute to lower frequencies of the resistant pathogen (Park et al., 2015) on pastures and agricultural landscapes.

## CONFLICT OF INTEREST

The authors declare no conflict of interest.

## ETHICS STATEMENT

The authors confirm that the ethical policies of the journal, as noted on the journal's author guidelines page, have been adhered to. No ethical approval was required, and ethical statement is not applicable as sample collection from animals has been gathered after animals were culled for management purposes according to the official culling plan to reduce red deer density that has been authorised by Istituto Superiore per la Protezione e la Ricerca Ambientale (ISPRA), the Italian Ministry of Environment (Prot. 48585/T‐A25‐Ispra), in the Lombardy sector of the Park starting from 2011. Therefore animals were not sacrificed for research purposes specific to this study.

## Supporting information

Data set 1. S. aureus isolates (n = 79) characterized using the StaphyType DNA microarray based assay

## Data Availability

The data that supports the findings of this study are available in the Supplementary Information of this article.
